# Identification and differentiation of *Campylobacter* isolated from chicken meat using real-time polymerase chain reaction and high resolution melting analysis of *hipO* and *glyA* genes

**DOI:** 10.14202/vetworld.2020.1875-1883

**Published:** 2020-09-15

**Authors:** Ika Kartika Syarifah, Hadri Latif, Chaerul Basri, Puji Rahayu

**Affiliations:** 1 Veterinary Public Health Study Program, Faculty of Veterinary Medicine, IPB University, Bogor, Indonesia; 2 Quality Control Laboratory and Certification of Animal Products, Bogor, Indonesia; 3 Department of Animal Infectious Diseases and Veterinary Public Health, Faculty of Veterinary Medicine, IPB University, Bogor, Indonesia

**Keywords:** *Campylobacter coli*, *Campylobacter jejuni*, chicken meat, *glyA*, *hipO*, melting curves

## Abstract

**Background and Aim:**

*Campylobacter* species have been recognized as the most frequently identified bacterial cause of human gastroenteritis. The aims of this study were to identify *Campylobacter jejuni* and *Campylobacter coli* species isolated from chicken meat and to analyze the differences in the melting curve patterns of both species.

**Materials and Methods:**

A total of 105 chicken meat samples collected from slaughterhouses and retailers in six provinces in Indonesia were examined for the isolation and identification of *Campylobacter* spp. A total of 56 positive isolates of *Campylobacter* spp. were analyzed using the quantitative real-time polymerase chain reaction and high resolution melting method.

**Results:**

The prevalence of *Campylobacter* spp. in chicken meat was found to be 61.9%. Regarding the identification, 23 isolates (41.07%) were *C. jejuni*, 22 (39.29%) were *C. coli*, six (10.71%) were a mix between *C. jejuni* and *C. coli*, and five isolates (8.93%) were *Campylobacter* spp. All the *C. jejuni* and *C. coli* isolates produced varied melting curve patterns.

**Conclusion:**

The high prevalence of *C. jejuni* and *C. coli* in chicken meat in Indonesia indicates a high risk of the incidence of campylobacteriosis in humans.

## Introduction

*Campylobacter* spp. are the bacteria those cause foodborne disease. They are the leading cause of acute gastroenteritis in humans and have an impact on public health. Cases of infection caused due to these bacteria pose a significant economic burden. It has been observed that the incidence and prevalence of campylobacteriosis have increased in both developed and developing countries over the past 10 years [1]. These bacterial infections cause diarrhea (sometimes bloody diarrhea), abdominal pain, fever, and complications that can lead to Guillain–Barré syndrome, reactive arthritis, and inflammatory bowel diseases [2]. Campylobacteriosis in humans is primarily caused due to two species, namely, *Campylobacter jejuni* and *Campylobacter coli* [3]. *C. jejuni* is responsible for causing 81% of campylobacteriosis incidence in humans, whereas 8.4% of the incidence is caused due to *C. coli*, and the remaining 10.6% is caused due to *Campylobacter lari*, *Campylobacter fetus*, and *Campylobacter upsaliensis* [1]. Although reports of gastroenteritis caused due to *Campylobacter* spp. are rare, especially in poor and developing countries, studies conducted in developed countries have estimated an incidence of 4.4-9.3 per thousand population per year [2]. The Centers for Disease Control and Prevention estimated that approximately 9% of foodborne diseases in the United States are caused due to *Campylobacter* spp. and as much as 15% of campylobacteriosis cases required intensive care in hospitals [4].

Chicken meat and other poultry meats are the major sources of campylobacteriosis. *Campylobacter* spp. can colonize asymptomatically in chickens and are considered as commensal gastrointestinal microbiota [5]. The process of slaughtering chickens in poultry slaughterhouses creates the opportunity for cross-contamination and bacterial spread even though the hygiene of the process is well maintained [6]. Chicken meat obtained from an uninfected farm can be contaminated with *Campylobacter* from another farm previously slaughtered at the same poultry slaughterhouse. Research conducted in China shows a very high prevalence of 80% of *Campylobacter* when slaughtering poultry [7]. Routine testing for pathogenic bacteria that cause foodborne diseases such as campylobacteriosis in the food of animal origin is a critical component of food safety management. The prevalence and contamination level of *Campylobacter* spp. in slaughterhouses and retailers can be used in the implementation of food safety policies and the evaluation of strategies to minimize risks to consumers [8,9].

Analysis conducted using real-time polymerase chain reaction and high resolution melting (qPCR-HRM) could be an efficient and robust molecular method to distinguish variations in DNA sequences [10]. HRM is a method that involves amplification in the presence of a saturation dye using PCR, and the subsequent melting of the amplicons gradually results from an increase in temperature. The melting pattern results indicate the characteristics of the DNA formed [11]. Numerous studies in the food safety sector have demonstrated that qPCR-HRM analysis can be applied for screening genotypes and variants based on the melting points of DNA fragments of pathogenic foodborne bacteria [8,12]. The HRM technique has been used for genotyping *C. jejuni* and *C. coli* to detect and differentiate between the two species on the basis of visual interpretation of differences in the melting curve patterns [9].

Therefore, the aims of this study were to identify the species of *C. jejuni* and *C. coli* isolated from chicken meat in Indonesia and to analyze the differences in the shape of the melting curves of both species.

## Materials and Methods

### Ethical approval

Ethical approval was not required in this study. However, samples were collected as per the standard sample collection procedure.

### Study period and location

The study was conducted from January 2018 to November 2019 at Quality Control Laboratory and Certification of Animal Products, Ministry of Agriculture, Republic of Indonesia.

### Campylobacter spp. isolate samples

Chicken meat samples obtained from the Monitoring and Surveillance Program of Animal Product run by the Quality Control Laboratory and Certification of Animal Products, Ministry of Agriculture, Republic of Indonesia, were used in this study. These samples were collected from the provinces of Aceh, Lampung, Banten, Jakarta, West Java, and Central Java. The samples were collected from the poultry slaughterhouses or retailers that already possessed a Veterinary Establishment Certificate, and sampling was conducted from January to December 2018.

A total of 105 samples were obtained from the program. The samples were initially tested by screening using the Singlepath Campylobacter (Merck, UK) rapid test. Positive test results from the rapid test were confirmed using a test method based on ISO 10272-1: 2006 concerning the microbiology of food and animal feedstuff – horizontal method for detection and enumeration of *Campylobacter* spp. – Part 1: Detection method. The *Campylobacter* spp. those were identified and suitable for storage were transferred to Brucella Broth (Merck, Germany) and glycerol (Merck, Germany) medium (20%) in cryotubes (Corning, USA) and then frozen at −20°C or −80°C for further extended storage.

### DNA extraction

The DNA from the isolates was extracted using the Mericon DNA Bacteria Kit (Qiagen, Germany), according to the manufacturer’s instructions. An amount of 0.2 mL of *Campylobacter* spp. isolate was taken from the cryotube and added to 0.8 mL of sterile phosphate-buffered saline (PBS) in a 2-mL microtube and then centrifuged at 11,900 rpm for 5 min. The resulting supernatant was removed using a pipette, and then 200 μL of sterile PBS was added to the bacterial pellet. The mixture was homogenized and centrifuged again at 11,900 rpm for 5 min. This procedure of washing the bacterial pellet was conducted until the suspension became colorless. The final step was the addition of 200 μL of Fast Lysis Buffer (Mericon DNA Bacteria Kit Qiagen, Germany). The suspension was placed in a ThermoMixer, heated at 100°C for 10 min at 800 rpm, and then incubated at room temperature for 2 min. The resulting suspension was centrifuged at 11,900 rpm for 5 min, and then 100 μL of the supernatant was transferred to a new 1.5-mL microtube and stored at −20°C or −80°C until further analysis.

### *Campylobacter* spp. isolate analysis using qPCR-HRM

The qPCR-HRM test protocol was implemented based on the procedure used by de Boer *et al*. [13] and modified using the intercalating dye SYBR Green Master Mix (Kapa Biosystems, USA). The primers used in this analysis were aimed to identify the *hipO* gene for *C. jejuni*, the *glyA* gene for *C. coli*, and the 16S rDNA *Campylobacter* gene for *Campylobacter* spp. Table-1 [13] shows the DNA sequences of the primer genes used to detect the *Campylobacter* isolates. The PCR mixture (20 μL) consisted of 25 μL of 10-μL SYBR Green Master Mix (Kapa Biosystems, USA), 1 μL (10 pmol) of the forward and reverse primers of each gene (Kapa Biosystems, USA), 3 μL of nuclease-free water, and 5 μL of DNA template.

**Table-1:** DNA sequences of the primers used for the detection of *Campylobacter* spp. isolates.

Primer name	Sequences (5’-3’)	Target	Reference
Cjejuni-F2	ATGAAGCTGTGGATTTTGCTAGTG	*hipO*	[13]
Cjejuni-R3	AAATCCAAAATCCTCACTTGCCA	*hipO*	
Ccoli-F2	CATATTGTAAAACCAAAGCTTATC	*glyA*	
Ccoli-R	AGTCCAGCAATGTGTGCAATG	*glyA*	
16S-CampyF1	CACGTGCTACAATGGCATATACA	16G rDNA *Campylobacter*	
16S-CampyR1	AAATCCAAAATCCTCACTTGCCA	16G rDNA *Campylobacter*	

The qPCR-HRM amplification was performed on a thermal cycler Rotor-Gene Q (Qiagen, Germany). The amplification program was conducted according to a modification of the method described by de Boer *et al*. [13]. Initial denaturation was performed at 95°C for 3 min, followed by 40 cycles of denaturation at 95°C for 3 s, annealing at 60°C for 30 s, and extension at 72°C for 20 s. The DNA melting program for HRM was based on Banowary *et al*. [9], where the measurements were conducted at a temperature of 70-90°C with a modification of the temperature increase speed of 0.1°C/s with a normalized region at 60-95°C for 5 s. The PCR analysis and the melting curve profile analysis were conducted using the Q-Rex (Qiagen, Germany) software.

All data obtained from the results of this study were analyzed descriptively. The differences in the melting curve patterns and the melting peak temperatures between the species in this study were analyzed and compared with those of *C. jejuni* ATCC 33291 (Microbiologic, France) and *C. coli* ATCCC 43478 (Microbiologic, France) as positive control references. Temperature standards for positive controls had been previously optimized (data not shown), and the melting peak temperatures were 74.5°C±0.1°C and 78.4°C±0.1°C for *C. jejuni* and *C. coli* positive controls, respectively.

All the obtained data were differentiated based on the *C. jejuni* isolates that produced melting curve patterns differing from those of the positive control and had a melting peak temperature other than 74.5°C±0.2°C, whereas for the *C. col*i isolates that produced melting curve patterns differing from those of the positive control and had melting peaks other than 78.4°C±0.2°C. This differentiation was based on Merchant-Patel *et al*. [14], who stated that HRM curves could be discriminated on the basis of obvious differences in the curve shape and/or on the basis of Tm, with a difference of 0.2°C being considered to be significant.

## Results

### Species identification of *Campylobacter* spp.

A total of 65 of the 105 chicken samples identified using the culture method showed positive results for *Campylobacter* spp., indicating a prevalence of 61.9%. The majority of positive bacterial-contaminated results exceeded 50% of the total samples collected in each province. Lampung and Jakarta were the provinces with the highest prevalence of 80% of *Campylobacter* spp., whereas Central Java Province had the least prevalence of 30%. A complete description of these results is provided in Table-2.

**Table-2:** The number of samples that were *Campylobacter* spp. positive from chicken meat samples in 2018 at the quality control laboratory and certification of animal products.

Province	Sample code	Total number of samples (n=105)	*Campylobacter* spp. Positive (n=65)	Percentage of *Campylobacter* positive samples (%)
Aceh	AC	20	10	50
Central Java	JTG	10	3	30
Lampung	LMP	20	16	80
Jakarta	JKT	10	8	80
West Java	JBR	20	11	55
Banten	BNT	25	17	68

The positive chicken samples yielded 56 isolates that could be analyzed for species identification. The *Campylobacter* spp. isolates were confirmed using the qPCR-HRM method for the identification of *C. jejuni* and *C. coli* species. On the basis of these results, the identification of the *Campylobacter* spp. isolates indicated that 23 isolates (41.07%) were *C. jejuni* and 22 isolates (39.29%) were *C. coli*. Furthermore, there were six isolates (10.71%) that were a mix between *C. jejuni* and *C. coli*, and the remaining five isolates (8.93%) were not identified as either but were identified as *Campylobacter* spp.

The variation in the number of *Campylobacter* bacterial species in each province was different. The samples from Lampung and Banten Provinces were dominated by *C. jejuni* species, whereas *C. coli* species were predominant in samples collected from Central Java and Jakarta. The samples collected from West Java showed an equal number of *C. jejuni* and *C. coli* species; however, in Lampung Province, the samples were dominated by another species of *Campylobacter*. Table-3 shows the identification results.

**Table-3:** Species identification results of *Campylobacter* spp. isolates using qPCR-HRM.

Province	Total number of isolates	*C. jejuni* (%)	*C. coli* (%)	*Campylobacter* spp. (%)	Mix of *C. jejuni* and *C. coli* (%)
Aceh	10	2 (20)	1 (10)	5 (50)	2 (20)
Central Java	3	0 (0)	3 (100)	0 (0)	0 (0)
Lampung	15	9 (60)	4 (26.67)	0 (0)	2 (13.33)
Jakarta	7	2 (28.57)	5 (71.43)	0 (0)	0 (0)
West Java	11	5 (45.45)	5 (45.45)	0 (0)	1 (9.1)
Banten	10	5 (50)	4 (40)	0 (0)	1 (10)
Total (%)	56 (100)	23 (41.07)	22 (39.29)	5 (8.93)	6 (10.71)

qPCR-HRM=real-time polymerase chain reaction and high resolution melting, *C. jejuni=Campylobacter jejuni, C. coli=Campylobacter coli*

### Melting curve variations of *C. jejuni* and *C. coli*

**Figure-1:**
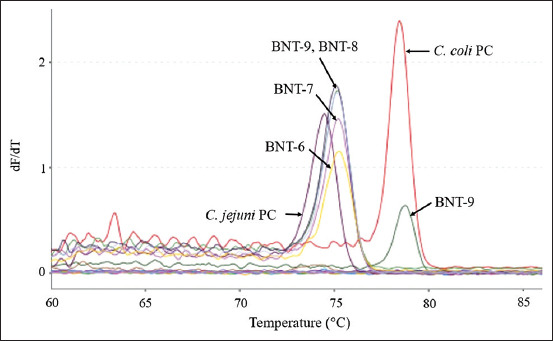
The melting curves of *Campylobacter jejuni* and *Campylobacter coli* isolates from Banten were tested using real-time polymerase chain reaction and high resolution melting. The left side is the melting curve of the *C. jejuni* (BNT-6, BNT-7, BNT-8, BNT-9, and *C. jejuni* positive control [PC]) isolates, while the right side is the melting curve of the *C. coli* (BNT-9 and *C. coli* PC) isolates.

All the *Campylobacter* spp. isolates tested in this study were analyzed descriptively based on the obtained visible melting curve patterns and melting peak temperatures, as mentioned in the materials and methods section. Overall, this method can detect the pattern of the melting curve of each isolate with two different temperature ranges that can demonstrate the differences between *C. jejuni* and *C. coli* species in one running PCR test, as depicted in Figure-1.

In the present study, 18 isolates of *C. jejuni* were found to have different melting curve patterns and melting peak temperatures, and five isolates had similar melting curve patterns and melting peak temperatures as those of the positive control. Meanwhile, there were 13 isolates of *C. coli* that had different melting curve patterns and melting peak temperatures, and there were nine isolates of *C. coli* that had similar melting curve patterns and peak temperatures as those of the positive control. These results are described in Table-4.

**Table-4:** The total number of *C. jejuni* and *C. coli* isolates distinguished by melting curves and melt peak temperatures compared to the positive control.

*Campylobacter* species	Melting curve pattern	

	Similar	Different
*C. jejuni* (n=23)	5	18
*C. coli* (n=22)	9	13
Mix of *C. jejuni* and *C. coli* (n=6)	2	4

C. jejuni=Campylobacter jejuni, C. coli=Campylobacter coli

**Figure-2:**
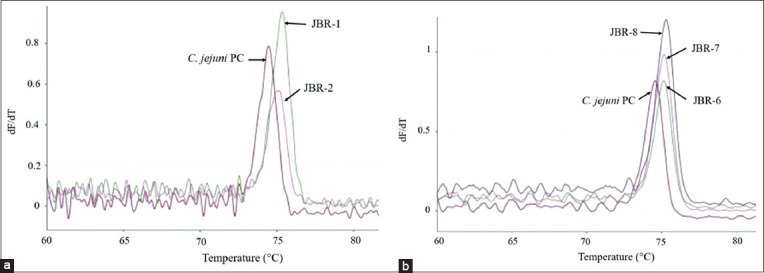
*Campylobacter jejuni* isolates from West Java (JBR) that showed variation in their melting curves. The melt peak temperatures for isolates JBR-1 and JBR-2 were 75.3°C and 74.9°C (a), JBR-6, JBR-7, and JBR-8 were 75.1°C (b), and the positive control for *C. jejuni* was 74.5°C.

**Figure-3:**
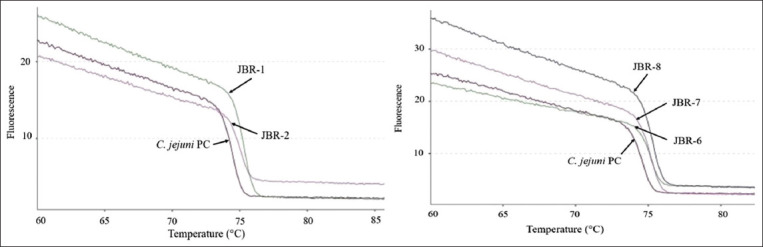
Normalized melting curve of *Campylobacter jejuni* isolates from West Java (JBR) and the *C. jejuni* positive control.

**Figure-4:**
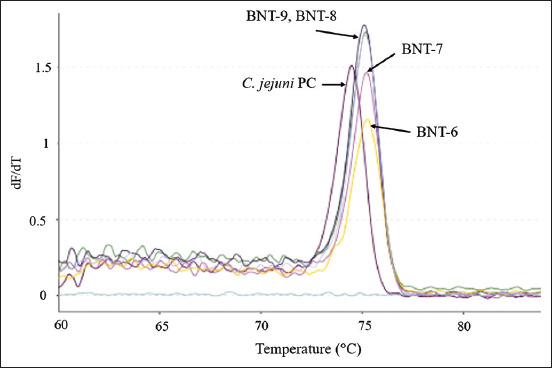
*Campylobacter jejuni* isolates from Banten (BNT) that showed variations in their melting curves. The melt peak temperatures for isolates BNT-6, BNT-7, and JBR-8 were 75.2°C, while BNT-9 was 75.1°C, and the positive control for *C. jejuni* was 74.5°C.

**Figure-5:**
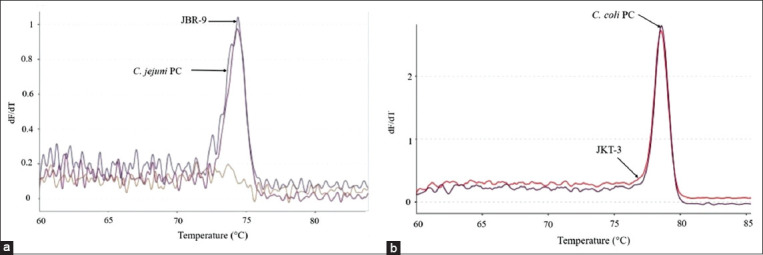
*Campylobacter jejuni* isolate from West Java Province (JBR) coded JBR-9 (a), and *Campylobacter coli* isolates from Jakarta Province (JKT) coded JKT-3 (b) showed similarities to the melting curve and melt peak temperature to the positive control.

Comparison of the melting curve patterns between the *C. jejuni* isolates and the positive control showed differences in isolates from Aceh, Lampung, Jakarta, West Java, and Banten Provinces. Similarity to the positive control was observed in isolates from Aceh, Lampung, and West Java Provinces. Variations based on the differences in melting curve patterns were detected in the *C. jejuni* isolates from West Java Province (JBR-1, JBR-2, JBR-6, JBR-7, and JBR-8). These results are depicted in Figures-2 and 3. The *C. jejuni* isolates from Banten Province (BNT-6, BNT-7, BNT-8, and BNT 9) also appeared to have different melting curve patterns compared with the positive control, as illustrated in Figure-4. Similar melting curve patterns as those of the positive control were detected in the *C. jejuni* isolates originating from West Java Province (JBR-9), as shown in Figure-5a.

Variations were also found in the different melting peak temperatures produced for each isolate. Differences based on the melting peak temperatures and melting curve patterns were found in isolates from West Java and Banten Provinces, as illustrated in Figures-2-4. The melting peak temperature for West Java isolates coded as JBR-1 and JBR-2 was 75.3°C and 74.9°C, respectively. The isolates coded as JBR-6, JBR-7, and JBR-8 had the same melting peak temperature of 75.1°C; the isolates from Banten coded as BNT 6, BNT-7, and BNT-8 also had the same melting peak temperature of 75.2°C, and that for the isolate coded as BNT-9 was 75.1°C.

**Figure-6:**
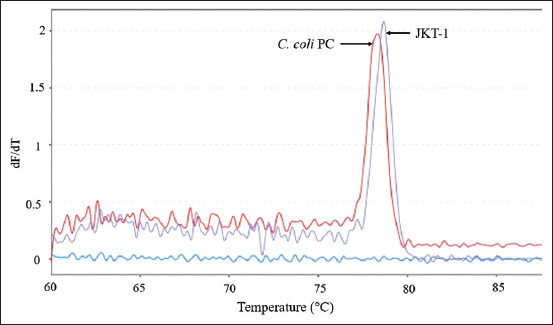
*Campylobacter coli* isolates from Jakarta Province (JKT) that showed variations in their melting curves. The melt peak temperature for isolate JKT-1 was 78.7°C, while the positive control for *C. coli* was 78.4°C.

*C. coli* isolates exhibiting differences and similarities in the melting curve patterns compared with the positive control were found in all six provinces. Variations in *C. coli* based on differences in the melting curve patterns were observed in isolates originating from Jakarta Province. This difference is shown in Figure-6 where the *C. coli* isolate (JKT-1) had a different melting curve pattern compared with the positive control. The similarity in the melting curve patterns with the *C. coli* positive control was also observed in an isolate originating from Jakarta Province (JKT-3) and is depicted in Figure-5b.

The variations in *C. coli* isolates were detected not only in the melting curve patterns but also in the melting peak temperatures produced for each isolate and were observed for isolates from Jakarta, as illustrated in Figure-6. The melting peak temperature of JKT-1 isolate was 78.7°C. Table-5 shows the results of species identification and melting peak temperatures.

**Table-5:** Species identification and melt peak temperature of *C. jejuni* and *C. coli* isolates using qPCR-HRM.

Isolate ID	Melt peak (°C)	Species identification	Isolate ID	Melt peak (°C)	Species identification
AC-3	74.3	*C. jejuni*	JKT-4	74.9	*C. jejuni*
AC-5	75.0	*C. jejuni*	JKT-5	79.0	*C. coli*
AC-8	74.4 and 79.0	*C. jejuni* and *C. coli*	JKT-6	78.7	*C. coli*
AC-9	74.1 and 78.6	*C. jejuni* and *C. coli*	JKT-7	75.0	*C. jejuni*
AC-10	78.6	*C. coli*	JBR-1	75.3	*C. jejuni*
JTG-1	78.8	*C. coli*	JBR-2	74.9 and 78.3	*C. jejuni* and *C. coli*
JTG-2	78.8	*C. coli*	JBR-3	78.4	*C. coli*
JTG-3	78.1	*C. coli*	JBR-4	78.7	*C. coli*
LMP-1	74.5 and 78.7	*C. jejuni* and *C. coli*	JBR-5	78.2	*C. coli*
LMP-2	78.9	*C. coli*	JBR-6	75.1	*C. jejuni*
LMP-3	78.7	*C. coli*	JBR-7	75.1	*C. jejuni*
LMP-4	78.9	*C. coli*	JBR-8	75.1	*C. jejuni*
LMP-5	74.9 dan 78.9	*C. jejuni* and *C. coli*	JBR-9	74.4	*C. jejuni*
LMP-6	75.4	*C. jejuni*	JBR-10	78.8	*C. coli*
LMP-7	74,9	*C. jejuni*	JBR-11	78,6	*C. coli*
LMP-8	74.8	*C. jejuni*	BNT-1	78,7	*C. coli*
LMP-9	74.5	*C. jejuni*	BNT-2	78.9	*C. coli*
LMP-10	74.9	*C. jejuni*	BNT-3	78.4	*C. coli*
LMP-11	74.7	*C. jejuni*	BNT-4	78.6	*C. coli*
LMP-12	75.2	*C. jejuni*	BNT-5	75.0	*C. jejuni*
LMP-13	74.7	*C. jejuni*	BNT-6	75.2	*C. jejuni*
LMP-14	78.4	*C. coli*	BNT-7	75.2	*C. jejuni*
LMP-15	75.4	*C. jejuni*	BNT-8	75.2	*C. jejuni*
JKT-1	78,7	*C. coli*	BNT-9	75.1 and 78.4	*C. jejuni* and *C. coli*
JKT-2	78.8	*C. coli*	BNT-10	75.0	*C. jejuni*
JKT-3	78.5	*C. coli*	ATCC 43478	78.4±0.1	*C. coli*
ATCC 33291	74.5±0.1	*C. jejuni*			

qPCR-HRM=real-time polymerase chain reaction and high resolution melting, *C. jejuni=Campylobacter jejuni, C. coli=Campylobacter coli*

## Discussion

### Prevalence of *Campylobacter* spp. in chicken meat samples

Campylobacteriosis has now been declared as one of the leading bacterial causes of human gastroenteritis in both developing and developed countries [1,2]. Several studies have related the high risk of campylobacteriosis in humans to chicken meat contaminated with *Campylobacter* [15]. In the present study, the prevalence of *Campylobacter* spp. originating from chicken meat in Indonesia was found to be 61.9% (65/105 chicken meat samples).

In a previous study, Zhang *et al*. [7] reported a high prevalence of 87.5-100% of *Campylobacter* contamination in samples collected from farms and in chilled chicken meat in China. Defeathering and evisceration are considered as critical points for cross-contamination in the process of slaughtering poultry [16,17]. Routine testing for *Campylobacter* on farms before the chickens are sent to the slaughterhouses or after slaughtering is essential to reduce these risks [18].

### Species identification of the Campylobacter spp. isolates

Species identification of the 56 isolates of *Campylobacter* spp. using qPCR-HRM demonstrated a slightly higher proportion of *C. jejuni* species (41.07%) than *C. coli* species (39.29%). There was a small proportion (10.71%) of isolates that was a mix of both species. These results are consistent with those reported in the Netherlands [13], who mentioned that the number of *C. jejuni* and *C. coli* species was almost similar. In another study conducted in Sichuan, China [18], it was observed that there were isolates of *Campylobacter* spp. that might contain either *C. jejuni* or *C. coli*.

Based on several other studies such as those conducted in the United States of America [19], Canada, Belgium, Australia, the United Kingdom, Japan, and Indonesia [20,21]. *C. jejuni* was more commonly isolated from chickens than *C. coli* although the ratio differed among countries. However, in Ecuador, South Africa, and Thailand, *C. coli* was the dominant species found in chickens [21,22].

The transmission routes of campylobacteriosis in humans generally involve handling and consuming food or water contaminated with *Campylobacter* [2]. Some studies have suggested that although *Campylobacter* spp. are extremely sensitive to various stress conditions, *C. jejuni* can survive in various types of environments by forming biofilms [23,24] and can protect itself from chemical products, physical cleaning processes, and other types of environmental stress [25].

*C. jejuni* can survive in water for longer periods than *C. coli*. Therefore, *C. jejuni* has a greater opportunity to contaminate food and can infect animals and humans [26]. This has been supported by the statements issued by EFSA and ECDC [1], which confirmed that *C. jejuni* species are responsible for causing the majority of campylobacteriosis cases in humans compared to those caused by *C. coli*.

In a recent study conducted by Johansson *et al*. [24], it was observed that *C. coli* clade 3 isolates exhibited a potent cytotoxic effect on HT-29 cells (human colon cancer cells), which caused rapid cell death in the digestive tract, but this was not found for *C. coli* clades 1 or 2 and *C. jejuni*. These findings indicate that although *C. jejuni* has a greater potential to cause campylobacteriosis in humans than *C. coli*, the presence of *C. coli* clade 3, which is more virulent, causes both these species to pose similarly high risks in humans.

### Melting curve variations of the *Campylobacter* spp. isolates

The results of the qPCR-HRM analysis conducted using the *hipO* and *glyA* genes revealed variations in the melting curve patterns observed for in *C. jejuni* and *C. coli* isolates, as illustrated in Figures-1-6. De Boer *et al*. [13] identified *C. jejuni* and *C. coli* in some samples by real-time PCR using the *hipO* and *glyA* genes. Although this method could identify the two species without the need for media or further testing processes, it could not demonstrate the melting curve patterns and the melting peak temperatures.

Banowary *et al*. [9] conducted identification and differentiation study of *Campylobacter* isolates using multiplex PCR and HRM using the *asp* and *hipO* genes. This method identified and differentiated *C. jejuni* and *C. coli* species with a sensitivity and specificity of 100% and 92%, respectively. Moreover, this method was able to reveal the differences in intraspecies DNA sequence variations based on melting curve patterns, differences in melting peak temperatures, and genotyping confidence percentage. Gago *et al*. [27] also reported that the HRM method could distinguish between *Cryptococcus neoformans* var. *grubii* and *C. neoformans* var. *neoformans* based on the different melting curve patterns.

DNA characterization using the HRM method is achieved based on the length of the sequence, the primary content of guanine and cytosine, and the complementarity of DNA sequences. This method is highly sensitive in detecting changes in one nucleotide base and would produce a different melting curve pattern [11].

According to the study of Banowary *et al*. [9], the difference in melting curve patterns produced by the HRM method is a reflection of the variations in the DNA sequence of the target genes in each isolate by confirming the results using sequencing methods. The results demonstrated nucleotide sequence variations of the isolates consisting of nucleotide deletions, insertions, and substitutions in the *hipO* and *asp* genes.

The method of sequencing is believed to be the gold standard for detecting DNA sequence variations. However, in a meta-analysis, the HRM method is considered to be one of the preferred methods for detecting sequence variations among the currently available techniques. This is the first step before deciding to continue using the sequencing method, which is relatively expensive and more time-consuming [28]. The major advantages of using the HRM method are that it is relatively rapid and of low cost, uses generic instruments, and provides good results. The differentiation of isolates based on the variations in melting curve patterns does not require the sequencing method if it is used only to identify variations in the DNA sequences [29,30].

The differences in DNA sequences can have an enormous impact on the virulence and characteristics of bacterial strains [31]. The differences in the DNA sequence of *C. jejuni* can affect the expression of genes encoding flagellar glycosylation, the capsular outer membrane, biosynthesis of enzymes, which are the strategies used by the bacterium for survival [32]. *C. jejuni* and *C. coli* have very similar phenotypic and genotypic characteristics that are difficult to distinguish [10]. Based on the present study, the identification and differentiation of *C. jejuni* or *C. coli* can be made with qPCR-HRM using the *hipO* and *glyA* genes. Moreover, this method can reveal the melting curve pattern variations and melting peak temperatures for each species.

## Conclusion

The majority of chicken meat samples collected from six provinces in Indonesia were contaminated with *Campylobacter* spp. (61.9%). The species most commonly found were *C. jejuni* and *C. coli* with almost similar percentages. A small proportion of isolates were a mix between *C. jejuni* and *C. coli*. These *C. jejuni* and *C. coli* isolates showed variations in the melting curve patterns and melting peak temperatures produced using the qPCR-HRM method. The high prevalence of *C. jejuni* and *C. coli* in chicken meat in Indonesia indicates a high risk of campylobacteriosis incidence in humans.

## Authors’ Contributions

IKS designed the study, performed the test, data analysis and drafted the manuscript under the supervision of HL, CB and PR. All authors read and approved the final manuscript.
